# Towards an accurate description of perovskite ferroelectrics: exchange and correlation effects

**DOI:** 10.1038/srep43482

**Published:** 2017-03-03

**Authors:** Simuck F. Yuk, Krishna Chaitanya Pitike, Serge M. Nakhmanson, Markus Eisenbach, Ying Wai Li, Valentino R. Cooper

**Affiliations:** 1 Materials Science and Technology Division, Oak Ridge National Laboratory, Oak Ridge, TN 37831, USA; 2 Department of Materials Science & Engineering, and Institute of Materials Science, University of Connecticut, Storrs, Connecticut 06269, USA; 3 National Center for Computational Sciences, Oak Ridge National Laboratory, Oak Ridge, TN 37831, USA

## Abstract

Using the van der Waals density functional with C09 exchange (vdW-DF-C09), which has been applied to describing a wide range of dispersion-bound systems, we explore the physical properties of prototypical *AB*O_*3*_ bulk ferroelectric oxides. Surprisingly, vdW-DF-C09 provides a superior description of experimental values for lattice constants, polarization and bulk moduli, exhibiting similar accuracy to the modified Perdew-Burke-Erzenhoff functional which was designed specifically for bulk solids (PBEsol). The relative performance of vdW-DF-C09 is strongly linked to the form of the exchange enhancement factor which, like PBEsol, tends to behave like the gradient expansion approximation for small reduced gradients. These results suggest the general-purpose nature of the class of vdW-DF functionals, with particular consequences for predicting material functionality across dense and sparse matter regimes.

*AB*O_*3*_ perovskite oxides, such as PbTiO_3_ and Pb(Zr, Ti)O_3_, are an important family of multifunctional compounds due to their high dielectric and piezoelectric responses^1^,^2^,^3^. These materials are attractive for numerous technological applications such as electronics, catalysis, superconductors, and electromechanical energy conversion^4^,^5^. Recently, major efforts have been invested in designing new lead-free piezoelectric oxides in order to minimize environmental damage^6^,^7^,^8^,^9^,^10^. First-principles methods based on density functional theory (DFT) have been routinely used to explore the ferroelectric phase transition of transition metal oxides at the atomistic level. A key challenge is the fact that complex behavior such as order-disorder transitions and domain wall motion typically require a large number of atoms in order to adequately simulate this behavior. Thus, some of the most common tactics for efficiently studying ferroelectric phase transitions include: (1) Monte Carlo simulations employing DFT parameterized effective Hamiltonians^11^,^12^,^13^,^14^,^15^,^16^,^17^ or (2) molecular dynamic simulations with atomistic potentials, such as the shell-model^18^,^19^,^20^ or bond-valence^21^,^22^,^23^ approach. While the above methods correctly reproduce the overall sequence of phase transitions for various perovskite oxides, the values of transition temperatures were generally underestimated relative to the experiments^11^,^12^,^24^. Such discrepancies in transition temperatures is likely caused by a combination of issues including an insufficient description of thermal expansion effects, failure to capture the important cooperative effects of ferroelectric distortions, and/or the error associated with the choice of DFT exchange-correlation functional^25^,^26^,^27^,^28^,^29^.

It is known that the practical error of DFT is closely related to its treatment of exchange and correlation effects. The binding energy between atoms tends to be overestimated by the local density approximation (LDA), resulting in an underestimation of optimized lattice parameters, particularly in perovskite oxides^30^,^31^. In contrast, the generalized gradient approximation (GGA) overcorrects the LDA errors, resulting in an overestimation of lattice parameters. Due to the strong volume dependence of structural instabilities in ferroelectric materials^31^,^32^,^33^,^34^,^35^,^36^,^37^, even modest errors in the values of their lattice parameters may sometimes lead to sizeable inaccuracies in predicting their functional properties with DFT. To overcome this issue, numerous approaches have been proposed to improve the exchange and correlation part of the density functional. Wu and Cohen modified the GGA functional (WC-GGA) by tuning the exchange enhancement factor of Perdew-Burke-Erzenhoff (PBE), which resulted in a better description of ferroelectric PbTiO_3_ and BaTiO_3_ phases compared to GGA and meta-GGA^38^. A similar approach was also adopted by Perdew and co-workers for their modified PBE for solids (PBEsol) functional, which gives predicted lattice parameters in good agreement with experimental values for various materials^39^. Hybrid exchange-correlation functionals, such as HSE and B1-WC, have also been developed to provide accurate descriptions of structural and electronic structures of ferroelectric oxides^40^,^41^. More recently, the class of van der Waals density functionals (vdW-DF), which account for long-range dispersion interactions, has shown remarkable success in representing not only dispersion-bound, but also densely-packed systems, including ferroelectric bulk oxides and metals^42^,^43^,^44^,^45^,^46^,^47^,^48^,^49^,^50^,^51^,^52^,^53^,^54^.

In this paper, we study how the choice of exchange-correlation functionals affects the prediction of the structural and ferroelectric properties of prototypical PbTiO_3_ (PTO), BaTiO_3_ (BTO), and KNbO_3_ (KNO) perovskites. As expected, LDA underestimates both the lattice parameters and spontaneous polarizations, while PBE overestimates them. In contrast, we observed the opposite trend when evaluating the bulk modulus due to the inverse relationship between total energy and optimized volume. Overall, vdW-DF with C09 exchange (vdW-DF-C09), followed by PBEsol, provides the best agreement with the experimental values, demonstrating its utility as a general-purpose functional for studying ferroelectric perovskite oxides. Additional analyses of the double-well potential surfaces and phase transition temperatures clearly confirm that the choice of the exchange and correlation functional may significantly impact the values of predicted ferroelectric properties of perovskite oxides. Recent studies on the application of a similar functional, vdW-DF-cx, to various systems, ranging from the ferroelectric response in PTO to the adsorption of small molecules, further supports the robustness of such vdW-DF-type functionals^52^,^53^,^55^. The overall trends in the performance of different functionals can be strongly correlated with the behavior of the exchange enhancement factor in the small reduced gradient region.

## Computational Details

All DFT calculations were performed using the plane-wave-based Quantum Espresso software package^56^. The energy cutoff for the plane-wave basis set was 50 Ry (200 Ry for the charge density cutoff), along with ultrasoft pseudopotentials^57^ (scalar-relativistic for *A*- and *B*-site cations and non-relativistic for O) to represent the electron-ion interaction. This cutoff criterion has also been employed in high-throughput DFT calculations using the ultrasoft pseudopotential library proposed by Garrity, Bennett, Rabe, and Vanderbilt^58^. Ba (*5s, 5p, 5d, 6s, 6p*), K (*3s, 3p, 4s, 4p*), Nb (*4s, 4p, 4d, 5s, 5p*), Pb (*5d, 6s, 6p*), Ti (*3s, 3p, 3d, 4s*), and O (*2s, 2p*) were considered as valence orbitals. A Monkhorst-Pack *k*-point mesh of 4 × 4 × 4 was determined to be sufficient for sampling the Brillouin zone. The quasi-Newton BFGS algorithm was used to optimize the bulk geometry with a force convergence criterion of 3.0 × 10^−4^ Ry/Bohr. To compare the effects of various exchange-and-correlation parameterizations on the structural and ferroelectric properties of perovskite oxides, we considered four types of functionals: LDA^59^, PBE^60^, PBEsol^39^, and vdW-DF^43^,^44^ with C09 exchange (vdW-DF-C09)^47^.

A 2 × 2 × 2 unit cell, consisting of 40 atoms, was first constructed to represent the centrosymmetric bulk structure of cubic PTO, BTO, and KNO perovskites. Subsequent displacements of *B*-site cations along [001], [011], and [111] directions were then induced to create tetragonal, orthorhombic, and rhombohedral phases, respectively. The detailed structural information of each crystal phase for PTO, BTO, and KNO can be found in Table S1 of Supplementary Information. In all cases, the cell parameters were relaxed together with the ionic coordinates, using a stress convergence criterion of 0.5 kbar. The Berry phase approach^61^ was applied to calculate spontaneous polarizations, *P*, from the optimized bulk geometries. The bulk modulus, *B*, of each crystal phase was evaluated using the Birch-Murnaghan^62^ equation of state:


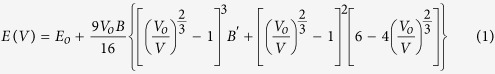


where *E*_*o*_ and *V*_*o*_ are the total energy and optimized volume of each phase, respectively. Thus, *B* and its first derivative, *B*′, can be computed by fitting the above equation with DFT energies and volumes near the energy minimum.

## Results and Discussion

### Influence of exchange-correlation functionals on structural properties

**Figure 1:**
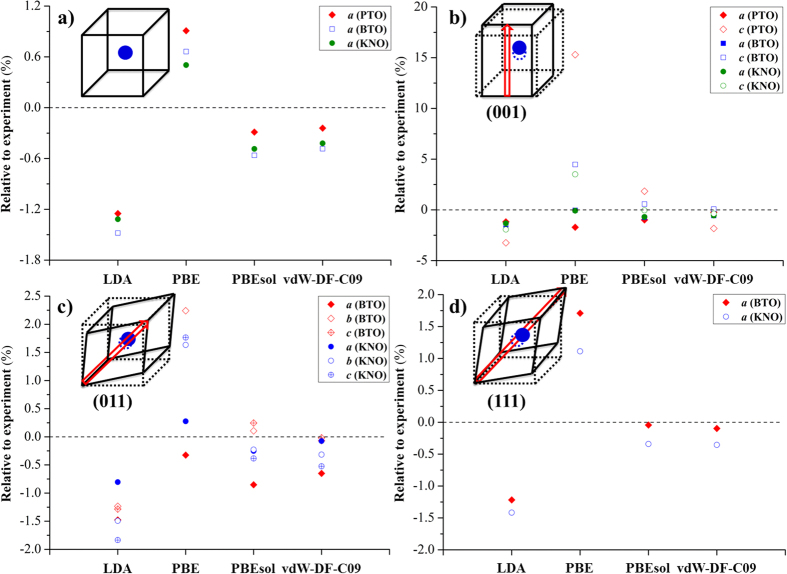
Percent error(%) of DFT lattice parameters evaluation for PTO, BTO, and KNO in the (**a**) cubic, (**b**) tetragonal, (**c**) orthorhombic, and (**d**) rhombohedral phases compared to the experimental values.

Figure 1 shows the percent error of DFT lattice parameters for PTO, BTO and KNO in different crystal phases relative to the experimental values (the magnitudes of the lattice parameters are presented in Table 1). As expected, LDA always underestimates the lattice parameters, while PBE overestimates them. In particular, there is a significant overestimation of the *c* lattice parameter (as large as ~15% deviation) in the tetragonal phase when using the latter. In contrast, PBEsol and vdW-DF-C09 predicted lattice parameters close to the experimental values (less than ~3% deviation).

**Figure 2:**
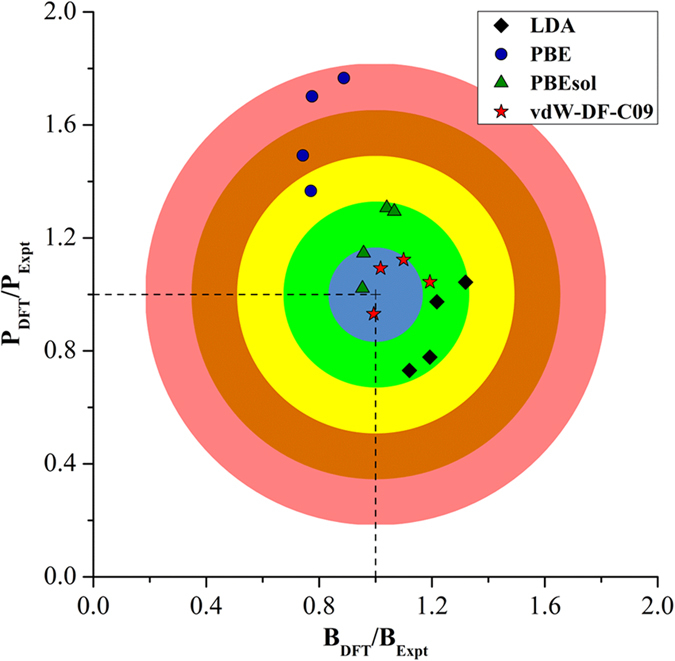
Comparison of bulk modulus (*B*_*DFT*_) and spontaneous polarizations (*P*_*DFT*_) of PTO, BTO, and KNO in the different crystal phases relative to the experimental values. Each color region represents the DFT/Experiment ratio of ±0.165.

As mentioned above, some of the most responsive piezoelectrics are perovskite oxides. Piezoelectricity is closely related to the following properties: the compressibility of the materials and the magnitude and orientation of polarization^63^,^64^,^65^. In this regard, correctly estimating the spontaneous polarization and elastic properties is the key to understanding phase transition temperatures and electromechanical coupling. Thus, it is an important task to study the exchange and correlation effects on the prediction of these properties for the accurate measurement of piezoelectric responses in perovskites. The bulk modulus and spontaneous polarizations of PTO, BTO, and KNO in different crystal phases were computed and compared to experiment (Table 1). Figure 2 depicts the ratio of the DFT spontaneous polarization (*P*_*DFT*_) relative to the experimental value (*P*_*Expt*_) as a function of the ratio of the DFT bulk modulus (*B*_*DFT*_) relative to experiment (*B*_*Expt*_). Again, using LDA and PBE leads to the under- and overestimation of spontaneous polarizations, respectively. The opposite trend was observed for the bulk modulus, generally with larger errors compared to the lattice parameters. The trend in bulk moduli is a direct consequence of the inverse dependence of total energy on optimized volume as shown in Equation (1). In other words, overestimations in volume are translated to underestimations in bulk moduli^66^. Overall, we found that vdW-DF-C09 produces the best agreement with experiments when predicting the structural properties of perovskite oxides, while PBEsol yields a slightly lower accuracy in property evaluations compared to the former. In fact, similar performance was also seen with vdW-DF-cx when predicting the material properties for cubic and tetragonal phase of PTO^52^. Thus, vdW-DF-C09 and PBEsol functionals should be suitable for further studies on the phase transitions of ferroelectric oxides.

**Figure 3:**
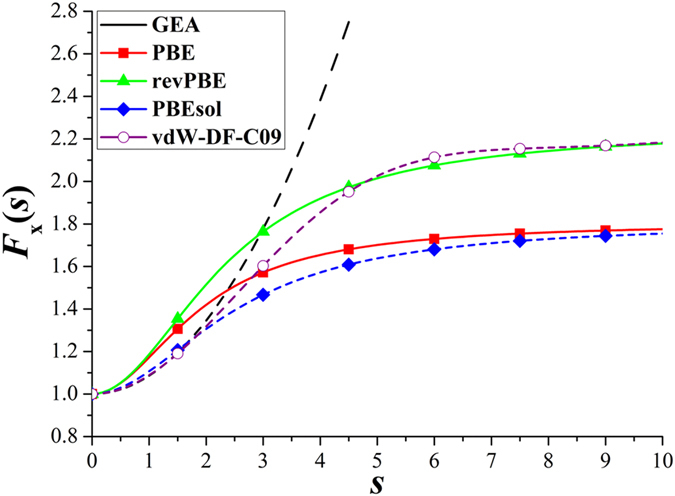
Exchange enhancement factor (*F*_x_(*s*)) for GEA, PBE, revPBE, PBEsol, and vdW-DF-C09 functionals.

A closer look at the exchange enhancement factor (*F*_x_(*s*)), which is a function of the reduced density gradient (*s* = ∇*ρ*/*ρ*), shows a clear difference in the overall trends with respect to the form of the exchange functionals, as displayed in Fig. 3. For a standard GGA, the exchange energy, *E*_x_(s), can be written as:


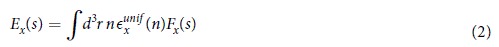


where 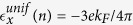 is the exchange energy per particle in a uniform gas with K_F_ = 3π^2^*n*. For *s* < 2, *F*_x_(*s*) of vdW-DF-C09 matches PBEsol, which tends to the form of the gradient expansion approximation (GEA)^67^; where F_x(s) = 1 + \mu s_2_ with \mu=0.0864. On the other hand, *F*_x_(*s*) of PBE significantly deviates from the GEA in the small s region. For large *s*, vdW-DF-C09 recovers the behavior of the revised PBE (revPBE)^68^ exchange, while PBEsol asymptotes to the PBE limit. Since reducing *F*_x_(*s*) for small *s* values leads to a decrease in short-range repulsion^39^,^47^, the treatment of *F*_x_(*s*), and thus, short-range repulsion by PBE leads to the overestimation of structural properties, while the opposite holds true for LDA (whose *F*_x_(*s*) = 1). *F*_x_(*s*) of PBEsol and vdW-DF-C09 sits between LDA and PBE and behaves similarly in the small *s* region, providing a more suitable prediction in structural properties compared to both LDA and PBE.

### Influence of exchange-correlation functionals on ferroelectric properties

**Figure 4:**
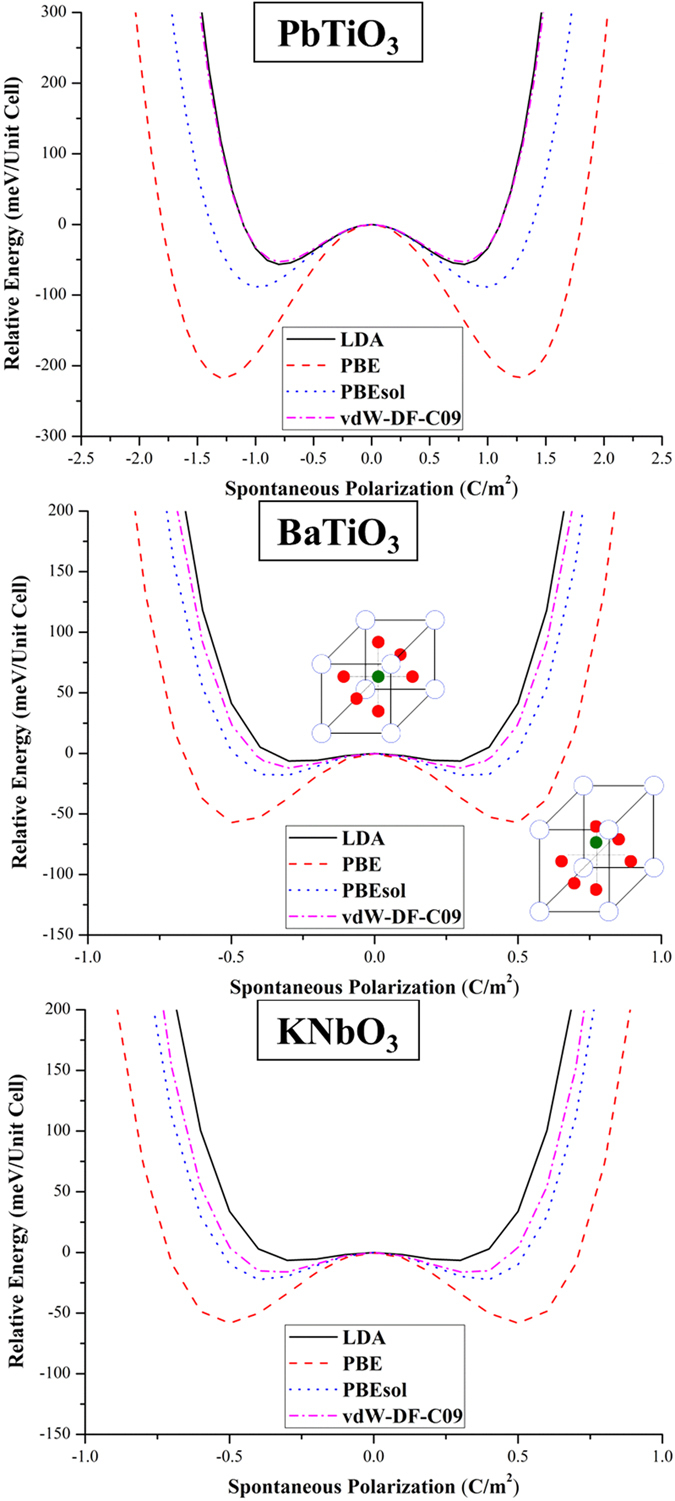
A double well potential for PTO, BTO, and KNO for cubic-to-tetragonal transition obtained with using LDA, PBE, PBEsol, and vdW-DF-C09. The fully optimized cubic structure was initially used for each perovskite. The lattice constants for the DFT optimized cubic structures are listed in the Supplementary information.

It is generally thought that the magnitude of the ferroelectric double well potential depth scales with the magnitude of the polarization. Changes in the predictions of ferroelectric well depths, consequently, may have large effects on predictions of ferroelectric phase transitions as well as responses to the application of an external electric field. The double well potential for the cubic-to-tetragonal transition can be expressed as a function of DFT-derived spontaneous polarizations based on the Landau theory (without considering strain couplings)^69^:


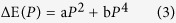


where ΔE is the total energy relative to the saddle point and *P* is the spontaneous polarization from DFT calculations. At the ferroelectric minimum, Equation (3) can be rearranged to find the value of constants: 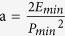 and 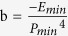. Greater potential well depths are expected for PTO, due to the presence of Pb lone pairs which results in a stronger hybridization between Pb 6*s* and O 2*p* orbitals resulting in larger polarizations in PTO as compared to BTO and KNO^33^. More importantly, we observed that the potential well depth increases by the following order of functionals regardless of the material: LDA ≤ vdW-DF-C09 < PBEsol < PBE, as presented in Fig. 4. This is consistent with the notion that the well depth scales with the magnitude of the polarization^40^,^70^. Thus, the energy barrier is changed, already hinting at a variation in phase transition behavior when using different exchange-correlation functionals.

Ultimately, we would like to understand the effects of exchange correlation functionals on dynamic phenomena such as phase transitions, domain wall motion and piezoelectric response; thus requiring the need to perform Monte Carlo or molecular dynamics simulations. However, as a first approximation it is possible to estimate phase transition temperatures (*T*_*c*_) from the cubic phase to the tetragonal phase in these materials using Landau theory. In this regards, Grinberg and Rappe demonstrated that there exists a strong dependence of *T*_*c*_ on the local structures of Pb- and Bi-based solid solutions^71^,^72^. Such dependence was expressed as a linear relationship between *T*_*c*_ and spontaneous polarization (*P*) in Landau theory as follows^69^:


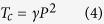


**Figure 5:**
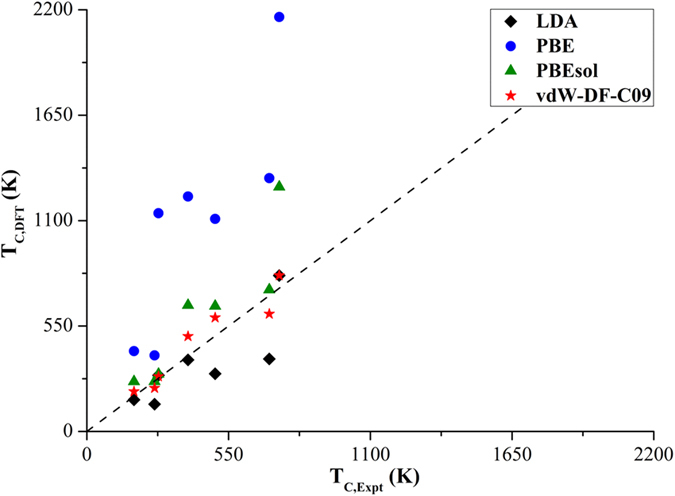
Comparison of phase transition temperatures (*T*_*C*,*DFT*_) of PTO, BTO, and KNO in the different crystal phases relative to the experimental values.

The constant *γ* of each crystal phase can be found by using the experimental spontaneous polarizations and temperatures. Thus, using such an approach, we can approximate *T*_*c*_ solely based on *P* from DFT calculations, as already summarized in Table 1. Of course, this approach cannot be used in a predictive sense as it requires experimental parameters to obtain the constant *γ*. Nevertheless, it emphasizes how small deviations in predictions of polarizations may be magnified in the prediction of other material behaviors. The estimated values of *T*_*c*_ from Equation (4) were determined over a large temperature range for different crystal phases of oxide perovskites, as shown in Fig. 5. Naturally, the phases with predicted *P* that are close to the experimental *P* yield the best agreement in *T*_*c*_ with experiment due to the nature of Equation (4). Again, the large variation in predicted temperatures is another indication that the exchange-correlation functional needs to be carefully chosen in order to accurately describe the ferroelectric phase transition of perovskite oxides.

## Conclusions

In conclusion, we studied how the choice of exchange-correlation functional influences both the structural and ferroelectric properties of prototypical PbTiO_3_, BaTiO_3_, and KNbO_3_ perovskites. As expected, under- and overestimations of lattice parameters and spontaneous polarizations were observed for LDA and PBE, respectively. In contrast, the opposite trend was seen in estimating the bulk modulus arising from the dependence of total energy on volume. Such variations in spontaneous polarizations and bulk moduli are a good (first) indication of exchange and correlation effects in predicting the responses in these materials. We found that a functional designed to describe sparse matter, vdW-DF-C09, gives the best agreement with experimental values for the structural properties. Similar performance was previously observed for a limited test of the vdW-DF-cx functional for the cubic and tetragonal phases of PTO. Both potential-well depths and transition temperatures were also greatly affected by choice of functionals. Detailed analyses of the exchange enhancement factor reveal that the variations in properties are strongly tied to the behavior of enhancement factor observed in the small *s* region. The PBEsol and C09 exchange functionals reproduce the form of GEA, thereby being more appropriate functionals when studying dense solids. It is worth noting that other exchange functionals that are often paired with the vdW-DF nonlocal correlation functional, including cx and optB86b, have similar forms in the small *s* region and may behave similarly for dense matter^53^,^54^. Overall, the selection of exchange-correlation functionals can induce non-negligible variations in predicting macroscopic properties of ferroelectric perovskite oxides, stressing the importance of exchange-correlation effects on such systems. In any event, these results provide benchmarks for the class of vdW-DF functionals that help to emphasize the role that the exchange functional plays in defining structural (and perhaps dynamic) properties of materials. Nevertheless, such considerations should be taken into account in the design or parameterization of new empirical/phenomenological models for studying dynamic and temperature- and pressure-dependent properties of bulk complex oxides.

## Additional Information

**How to cite this article:** Yuk, S. F. *et al*. Towards an accurate description of perovskite ferroelectrics: exchange and correlation effects. *Sci. Rep.* **7**, 43482; doi: 10.1038/srep43482 (2017).

**Publisher's note:** Springer Nature remains neutral with regard to jurisdictional claims in published maps and institutional affiliations.

## Supplementary Material

Supplementary Information

## Figures and Tables

**Table 1:** Structural and ferroelectric properties of PTO, BTO, and KNO in the different crystal phases using LDA, PBE, PBEsol, and vdW-DF-C09.

Phase		LDA	PBE	PBEsol	vdW-DF-C09	Expt.
**PbTiO**_**3**_						
Cubic	*a* (Å)	3.881	3.966	3.919	3.921	3.930^73^
	*B* (GPa)	205.27	173.21	190.08	190.76	195.00^74^
Tetragonal	*a* (Å)	3.858	3.837	3.865	3.895 (3.8955)^52^	3.904^75^
	*c* (Å)	4.017	4.787	4.228	4.076 (4.0788)^52^	4.152^75^
	*B* (GPa)	145.69	85.60	114.85	131.74	110.44^76^
	*P* (C/m^2^)	0.78	1.28	0.98	0.78 (0.75)^52^	0.75^77^
	*T*_*c*_ (K)	813	2162	1276	814	747^78^
**BaTiO**_**3**_						
Cubic	*a* (Å)	3.941	4.026	3.977	3.981	4.000^79^
	*B* (GPa)	195.96	162.65	179.25	178.65	195.00^80^
Tetragonal	*a* (Å)	3.933	3.991	3.962	3.969	3.992^81^
	*c* (Å)	3.980	4.216	4.059	4.039	4.036^81^
	*B* (GPa)	169.47	123.59	148.44	153.08	139.20^80^
	*P* (C/m^2^)	0.26	0.48	0.35	0.30	0.27^82^
	*Tc* (K)	373	1226	658	496	393^83^
Orthorhombic	*a* (Å)	3.931	3.977	3.956	3.964	3.990^84^
	*b* (Å)	5.599	5.796	5.675	5.665	5.669^84^
	*c* (Å)	5.609	5.848	5.696	5.681	5.682^84^
	*B* (GPa)	155.68	105.75	133.21	138.24	—
	*P* (C/m^2^)	0.31	0.61	0.31	0.30	0.30^82^
	*Tc* (K)	293	1139	299	287	278^83^
Rhombohedral	*a* (Å)	3.951	4.068	3.998	3.996	4.000^85^
	*B* (GPa)	151.17	95.95	121.73	129.36	—
	*P* (C/m^2^)	0.31	0.50	0.39	0.35	0.33^82^
	*T*_*c*_ (K)	165	419	260	208	183^83^
**KNbO**_**3**_						
Cubic	*a* (Å)	3.952	4.025	3.986	3.988	4.005^86^
	*B* (GPa)	204.27	176.77	189.62	185.49	—
Tetragonal	*a* (Å)	3.944	3.994	3.969	3.975	3.997^86^
	*c* (Å)	3.984	4.205	4.061	4.048	4.063^86^
	*B* (GPa)	184.85	127.17	157.36	163.93	165.00^87^
	*P* (C/m^2^)	0.27	0.51	0.38	0.34	0.37^88^
	*T*_*c*_ (K)	378	1322	739	613	708^89^
Orthorhombic	*a* (Å)	3.941	3.984	3.963	3.970	3.973^86^
	*b* (Å)	5.610	5.788	5.682	5.677	5.695^86^
	*c* (Å)	5.616	5.822	5.699	5.691	5.721^86^
	*B* (GPa)	174.15	108.42	139.82	148.63	146.00^90^
	*P* (C/m^2^)	0.32	0.61	0.47	0.45	0.41^91^
	*T*_*c*_ (K)	301	1109	654	594	498^89^
Rhombohedral	*a* (Å)	3.959	4.061	4.002	4.002	4.016^86^
	*B* (GPa)	168.67	101.61	136.40	141.67	—
	*P* (C/m^2^)	0.31	0.52	0.42	0.39	0.42^86^
	*T*_*c*_ (K)	142	397	261	226	263^89^

*B* and *P* are the bulk modulus and spontaneous polarization obtained for the optimized crystal structures, respectively. *T*_*c*_ represents the phase transition temperature of crystal phases predicted using Equation (4). Experimental values are provided for comparison.

Reference values in parentheses were obtained using the vdW-DF-cx functional^52^.
