# Erratum: Towards an accurate description of perovskite ferroelectrics: exchange and correlation effects

**DOI:** 10.1038/srep46647

**Published:** 2017-05-03

**Authors:** Simuck F. Yuk, Krishna Chaitanya Pitike, Serge M. Nakhmanson, Markus Eisenbach, Ying Wai Li, Valentino R. Cooper

Scientific Reports 7: Article number: 4348210.1038/srep43482; published online: 03 03 2017; updated: 05 03 2017

This Article contains errors. In the ‘Results and Discussion’ section,

“…the exchange energy per particle in a uniform gas with K_F_ = 3π^2^*n*. For *s* < 2, *F*_x_(*s*) of vdW-DF-C09 matches PBEsol, which tends to the form of the gradient expansion approximation (GEA)^67^; where F_x(s) = 1 + \mu s_2_ with \mu = 0.0864”.

should read:

“…the exchange energy per particle in a uniform gas with *k*_*F*_ = [3π^2^*n*]^1/3^. For *s* < 2, *F*_*x*_*(s)* of vdW-DF-C09 matches PBEsol, which tends to the form of the gradient expansion approximation (GEA)^67^; where *F*_*x*_(*s*) = 1 + *μ s*^*2*^ with *μ* = 0.0864”.

