# Supplementary material for: Towards an accurate description of perovskite ferroelectrics: exchange and correlation effects
Source: Sci Rep. 2017 Mar 3;7:43482. doi: 10.1038/srep43482 (PMC5335310; doi:10.1038/srep43482)
Supplement: Supplementary Information [file srep43482-s1.pdf]

Towards an accurate description of perovskite ferroelectrics: exchange and correlation effects

Simuck F. Yuk,[1] Krishna Chaitanya Pitike,[2] Serge M. Nakhmanson,[2] Markus Eisenbach,[3] Ying Wai Li,[3] and Valentino R. Cooper[1,*]

[1]Materials Science and Technology Division, Oak Ridge National Laboratory, Oak Ridge, TN 37831, USA
[2]Department of Materials Science & Engineering, and Institute of Materials Science, University of Connecticut, Storrs, Connecticut 06269, USA
[3]National Center for Computational Sciences, Oak Ridge National Laboratory, Oak Ridge, TN 37831, USA

Supplementary Information

The detailed structural information on each crystal phase of $PbTiO_3$, $BaTiO_3$, and $KNbO_3$ is summarized in Table S1, using LDA, PBE, PBEsol, and vdW-DF-C09 exchange-correlation functionals.

Table S1. Structural information (Wyckoff positions of atoms in fractional coordinates with respect to unit cells) of different crystal phases of $PbTiO_3$, $BaTiO_3$, and $KNbO_3$, using LDA, PBE, PBEsol, and vdW-DF-C09.

Cubic Phase (Pm-3m)

	LDA	PBE	PBEsol	vdW-DF-C09	Experiment
$PbTiO_3$:					
	**LDA**	**PBE**	**PBEsol**	**vdW-DF-C09**	**Experiment[1]**
a (Å)	3.881	3.966	3.919	3.921	3.930
$BaTiO_3$:					
	**LDA**	**PBE**	**PBEsol**	**vdW-DF-C09**	**Experiment[2]**
a (Å)	3.941	4.026	3.977	3.981	4.000
$KNbO_3$:					
	**LDA**	**PBE**	**PBEsol**	**vdW-DF-C09**	**Experiment[3]**
a (Å)	3.952	4.025	3.986	3.988	4.005

Tetragonal Phase (P4mm)

$PbTiO_3$:					
	**LDA**	**PBE**	**PBEsol**	**vdW-DF-C09**	**Experiment[4]**
a (Å)	3.858	3.837	3.865	3.895	3.904
c (Å)	4.017	4.787	4.228	4.076	4.152
**Atom**	Δz_{atom}	Δz_{atom}	Δz_{atom}	Δz_{atom}	Δz_{atom}
Pb: 1a (0, 0, 0)	---	---	---	---	---
Ti: 1b (0.5, 0.5, 0.5+Δz_{Ti})	0.033	0.056	0.040	0.031	0.0400
O_1: 1b (0.5, 0.5, Δz_{O1})	0.088	0.195	0.122	0.090	0.1120
O_2: 2c (0, 0.5, 0.5+Δz_{O2})	0.100	0.171	0.126	0.100	0.1120
$BaTiO_3$:					
	**LDA**	**PBE**	**PBEsol**	**vdW-DF-C09**	**Experiment[5]**
a (Å)	3.933	3.991	3.962	3.969	3.992
c (Å)	3.980	4.216	4.059	4.039	4.036
**Atom**	Δz_{atom}	Δz_{atom}	Δz_{atom}	Δz_{atom}	Δz_{atom}
Ba: 1a (0, 0, 0)	---	---	---	---	---
Ti: 1b (0.5, 0.5, 0.5+Δz_{Ti})	0.013	0.018	0.016	0.016	0.0203
O_1: 1b (0.5, 0.5, Δz_{O1})	-0.021	-0.052	-0.031	-0.025	-0.0259
O_2: 2c (0, 0.5, 0.5+Δz_{O2})	-0.013	-0.03	-0.019	-0.015	-0.0123
$KNbO_3$:					
	**LDA**	**PBE**	**PBEsol**	**vdW-DF-C09**	**Experiment[3]**
a (Å)	3.944	3.994	3.969	3.975	3.997
c (Å)	3.984	4.205	4.061	4.048	4.063
**Atom**	Δz_{atom}	Δz_{atom}	Δz_{atom}	Δz_{atom}	Δz_{atom}
K: 1a (0, 0, 0)	---	---	---	---	---
Nb: 1b (0.5, 0.5, 0.5+Δz_{Nb})	0.010	0.018	0.015	0.015	0.0230
O_1: 1b (0.5, 0.5, Δz_{O1})	-0.017	-0.045	-0.027	-0.022	-0.0170
O_2: 2c (0, 0.5, 0.5+Δz_{O2})	-0.018	-0.033	-0.024	-0.021	-0.0190

Orthorhombic Phase (Amm2)

$BaTiO_3$:

	**LDA**		**PBE**		**PBEsol**		**vdW-DF-C09**		**Experiment[6]**	
a (Å)	3.931		3.977		3.956		3.964		3.990	
b (Å)	5.599		5.796		5.675		5.665		5.669	
c (Å)	5.609		5.848		5.696		5.681		5.682	
**Atom**	Δy_{atom}	Δz_{atom}	Δy_{atom}	Δz_{atom}	Δy_{atom}	Δz_{atom}	Δy_{atom}	Δz_{atom}	Δy_{atom}	Δz_{atom}
Ba: 2a (0, 0, 0)	---	---	---	---	---	---	---	---	---	---
Ti: 2b (0.5, 0, 0.5+Δz_{Ti})	---	0.011	---	0.017	---	0.014	---	0.013	---	0.010
O_1: 2a (0, 0, 0.5+Δz_{O1})	---	-0.011	---	-0.023	---	-0.016	---	-0.013	---	-0.010
O_2: 4e (0.5, 0.25-Δy_{O1}, 0.75+Δz_{O2})	0.003	-0.013	0.009	-0.026	0.005	-0.018	0.004	-0.015	0.003	-0.013

$KNbO_3$:

	**LDA**		**PBE**		**PBEsol**		**vdW-DF-C09**		**Experiment[3]**	
a (Å)	3.941		3.984		3.963		3.970		3.973	
b (Å)	5.610		5.788		5.682		5.677		5.695	
c (Å)	5.616		5.822		5.699		5.691		5.721	
**Atom**	Δy_{atom}	Δz_{atom}	Δy_{atom}	Δz_{atom}	Δy_{atom}	Δz_{atom}	Δy_{atom}	Δz_{atom}	Δy_{atom}	Δz_{atom}
K: 2a (0, 0, 0)	---	---	---	---	---	---	---	---	---	---
Nb: 2b (0.5, 0, 0.5+Δz_{Nb})	---	-0.008	---	-0.016	---	-0.012	---	-0.012	---	-0.017
O_1: 2a (0, 0, 0.5+Δz_{O1})	---	0.016	---	0.026	---	0.020	---	0.018	---	0.004
O_2: 4e (0.5, 0.25-Δy_{O1}, 0.75+Δz_{O2})	0	0.013	-0.006	0.024	-0.002	0.018	-0.001	0.016	-0.013	0.018

Rhombohedral Phase (R3m)

$BaTiO_3$:

	**LDA**		**PBE**		**PBEsol**		**vdW-DF-C09**		**Experiment[7]**	
a (Å)	3.951		4.068		3.998		3.951		4.000	
**Atom**	Δx_{atom}	Δz_{atom}	Δx_{atom}	Δz_{atom}	Δx_{atom}	Δz_{atom}	Δx_{atom}	Δz_{atom}	Δx_{atom}	Δz_{atom}
Ba: 1a (0, 0, 0)	---	---	---	---	---	---	---	---	---	---
Ti: 1a (0.5+Δx_{Ti}, 0.5+Δx_{Ti}, 0.5+Δx_{Ti})	-0.009	-0.009	-0.015	-0.015	-0.012	-0.012	-0.011	-0.011	-0.013	-0.013
O_1: 3b (0.5+Δx_{O1}, 0.5+Δx_{O1}, Δz_{O1})	0.009	0.014	0.016	0.027	0.012	0.019	0.010	0.016	0.011	0.019

$KNbO_3$:

	**LDA**		**PBE**		**PBEsol**		**vdW-DF-C09**		**Experiment[3]**	
a (Å)	3.959		4.061		4.002		3.959		4.016	
**Atom**	Δx_{atom}	Δz_{atom}	Δx_{atom}	Δz_{atom}	Δx_{atom}	Δz_{atom}	Δx_{atom}	Δz_{atom}	Δx_{atom}	Δz_{atom}
K: 1a (0, 0, 0)	---	---	---	---	---	---	---	---	---	---
Nb: 1a (0.5+Δx_{Nb}, 0.5+Δx_{Nb}, 0.5+Δx_{Nb})	-0.007	-0.007	-0.013	-0.013	-0.011	-0.011	-0.010	-0.010	-0.011	-0.011
O_1: 3b (0.5+Δx_{O1}, 0.5+Δx_{O1}, Δz_{O1})	0.012	0.010	0.017	0.022	0.014	0.016	0.013	0.013	0.018	0.020

References

1. Mabud, S. A. & Glazer, A. M. Lattice parameters and birefringence in $PbTiO_3$ single crystals. *J. Appl. Crystallogr.* **12**, 49-53 (1979).
2. Hellwege, K. H. & Hellwege, A. M. *Ferroelectrics and Related Substances*. (Springer, 1969).
3. Hewat, A. W. Cubic-tetragonal-orthorhombic-rhombohedral ferroelectric transitions in perovskite potassium niobate: neutron powder profile refinement of the structures. *J. Phys. C: Solid State Phys.* **6**, 2559 (1973).
4. Shirane, G., Pepinsky, R. & Frazer, B. X-ray and neutron diffraction study of ferroelectric $PbTiO_3$. *Acta Crystallographica* **9**, 131-140 (1956).
5. Kwei, G. H., Lawson, A. C., Billinge, S. J. L. & Cheong, S. W. Structures of the ferroelectric phases of barium titanate. *J. Phys. Chem.* **97**, 2368-2377 (1993).

6. Shirane, G., Danner, H. & Pepinsky, R. Neutron Diffraction Study of Orthorhombic $BaTiO_3$. *Phys. Rev.* **105**, 856 (1957).
7. Hewat, A. W. Structure of rhombohedral ferroelectric barium titanate. *Ferroelectrics* **6**, 215-218 (1973).
